# The value of position-specific scoring matrices for assessment of protein allegenicity

**DOI:** 10.1186/1471-2105-9-S12-S21

**Published:** 2008-12-12

**Authors:** Shen Jean Lim, Joo Chuan Tong, Fook Tim Chew, Martti T Tammi

**Affiliations:** 1 Department of Biochemistry, Yong Loo Lin School of Medicine, National University of Singapore, 8 Medical Drive, Singapore 117597; 2 Institute for Infocomm Research, 21 Heng Mui Keng Terrace, Singapore 119613; 3 Department of Biological Sciences, National University of Singapore, 14 Science Drive 4, Singapore 117543; 4 Department of Microbiology, Tumor and Cell Biology, Karolinska Institutet, Stockholm 17177, Sweden

## Abstract

**Background:**

Bioinformatics tools are commonly used for assessing potential protein allergenicity. While these methods have achieved good accuracies for highly conserved sequences, they are less effective when the overall similarity is low. In this study, we assessed the feasibility of using position-specific scoring matrices as a basis for predicting potential allergenicity in proteins.

**Results:**

Two simple methods for predicting potential allergenicity in proteins, based on general and group-specific allergen profiles, are presented. Testing results indicate that the performances of both methods are comparable to the best results of other methods. The group-specific profile approach, with a sensitivity of 84.04% and specificity of 96.52%, gives similar results as those obtained using the general profile approach (sensitivity = 82.45%, specificity = 96.92%).

**Conclusion:**

We show that position-specific scoring matrices are highly promising for constructing computational models suitable for allergenicity assessment. These data suggest it may be possible to apply a targeted approach for allergenicity assessment based on the profiles of allergens of interest.

## Background

Atopic allergy and other forms of hypersensitivity reactions pose a major concern for public health, affecting up to 25% of the population in industrial nations [1,2]. With the rapid growth in the number of genetically modified (GM) food, biopharmaceuticals and other biotechnology-derived products, identifying potential allergenicity in proteins has become crucial in product safety assessment [3,4].

Unlike laboratory-based allergenicity assessment methods such as the skin prick test and RAST (radioallergosorbent test), which are often rigorous and time-consuming, the use of bioinformatics tools has come in favorably for accelerating the discovery of novel allergens. Guidelines to evaluate allergenicity potential of proteins have been jointly proposed by the Food and Agriculture Organization (FAO)/World Health Organization (WHO) Expert Consultation on Allergenicity of Foods Derived from Biotechnology [5]. According to the bioinformatics section of the guidelines, a protein is a potential allergen if it either has an identity of ≥ 6 continuous amino acids or ≥ 35% sequence similarity over a window of 80 amino acids with a known allergen.

Although useful in some cases, it has been shown that the FAO/WHO joint recommendation produces a large number of false positives, resulting in specificities that are too low to be of practical use [6,7]. To address these drawbacks, more sophisticated bioinformatics tools have been developed. These include support vector machines (SVM) [8], Gaussian classification algorithms [9,10], wavelet transform models [11], allergen motifs [12], IgE sequence comparisons [13,14] and the use of allergen-representative peptides (ARP) [15]. While these systems are effective for high similarity allergen sequences, they are less effective for when the overall similarity is low [16].

Position-specific scoring matrices (PSSM) have been very successful for detecting distantly related protein sequences [17-19], but have yet been applied for assessing allergenic potentials in proteins. In this study, we shall examine the feasibility of using PSSM as a basis for developing an effective allergenicity prediction system. As will be seen below, the use of an iterative PSI-BLAST in combination with various filters for accuracy optimization shows great promise for constructing general and group-specific profiles suitable for allergenicity assessment.

## Results and discussion

The performance of both profile-based approaches was evaluated using eight different E-value thresholds (Table 1). We consider values of SP ≥ 80% and SE ≥ 80% useful in practice [20] and assessed suitability of both methods using the above cutoffs.

**Table 1:** Prediction quality of the profile-based methods

**Method**	**E-value threshold**	**FN**	**FP**	**ACC (%)**	**SP (%)**	**SE (%)**	**PPV (%)**	**NPV (%)**	**MCC**
General Profile	10	11	1792	21.67	10.38	96.42	13.99	94.71	0.08
	1	22	737	67.03	63.17	92.58	27.74	98.25	0.38
	10^-1^	31	298	85.72	85.11	89.80	48.45	98.22	0.59
	10^-2^	36	184	90.42	90.80	87.95	60.22	98.03	0.68
	10^-3^	41	137	92.27	93.14	86.56	66.70	97.87	0.72
	10^-4^	43	108	93.44	94.61	85.70	71.76	97.77	0.75
	10^-6^	48	83	94.32	95.87	84.04	76.53	97.55	0.77
	10^-9^	53	62	95.02	96.92	82.45	81.34	97.34	0.79
Group-Specific	10	14	1801	21.14	9.92	95.43	13.79	93.48	0.06
Profiles	1	22	748	66.53	62.58	92.72	27.33	98.27	0.38
	10^-1^	29	317	84.99	84.17	90.40	46.88	98.31	0.58
	10^-2^	34	202	89.76	89.89	88.87	57.87	98.16	0.66
	10^-3^	37	151	91.83	92.44	87.81	64.73	98.04	0.71
	10^-4^	40	124	92.86	93.79	86.69	68.87	97.90	0.73
	10^-6^	44	94	94.02	95.31	85.50	74.51	97.75	0.76
	10^-9^	48	70	94.88	96.52	84.04	79.49	97.56	0.79

### General profile model

The predictive performance of the general allergen profile approach is in accordance with expected allergenic patterns in proteins and provided an accuracy (ACC) of greater than 85% (SE > 82%, SP > 85%) for E-value cutoffs of ≤ 10^-1^. This approach is shown to perform best at the E-value threshold of 10^-9 ^(ACC = 95.02%). At this threshold, the sensitivity and specificity of the model is 82.45% and 96.92% respectively.

### Group-specific profile model

Allergen sequences are currently classified into 9 major groups by the IUIS Allergen Nomenclature Sub-Committee  – i) weeds, ii) fungi, iii) grasses, iv) trees, v) mites, vi) animals, vii) insects, viii) food, and ix) others [21]. We constructed group-specific profiles based on all 9 major allergen groups, and tested their capability in predicting allergen sequences. As illustrated in Table 1, the approach achieved similar performance as the general profile model, and can predict allergens with high accuracy (ACC > 84%, SE > 84%, SP > 84%) at E-value thresholds of ≤ 10^-1^. The best performance is observed at the E-value threshold of 10^-9 ^(ACC = 94.88%). At this threshold, the sensitivity and specificity of the model is 96.52% and 84.04% respectively.

Next, we tested the ability of group-specific profiles in identifying allergens that belong to their respective group category (Table 2). Among the 9 group-specific profile models, 7 are capable of predicting allergens with accuracy greater than 80%. Mite profile model achieved the best performance with an accuracy of 95.29% (SE = 90.81%, SP = 95.80%), followed by grass profile model (ACC = 87.81%, SE = 87.16%, SP = 87.91%), and insect profile model (ACC = 87.20%, SE = 82.08%, SP = 87.82%). The poorest performance was observed for food model (ACC= 69.63%, SE = 83.22%, SP= 63.89%). This may be attributable to the fact that food allergens contain highly diverse protein sequences that do not share much common features and sequence patterns.

**Table 2:** Average prediction quality of the group-specific profiles. Performance of group-specific profile models at E-value threshold of 10^-9^.

**Profile**	**ACC (%)**	**SP (%)**	**SE (%)**	**PPV (%)**	**NPV (%)**	**MCC**
Animal	86.01	87.55	65.39	27.09	97.26	0.36
Food	69.63	63.89	83.22	48.85	90.37	0.43
Weed	77.79	78.44	69.33	18.70	97.26	0.27
Insect	87.20	87.82	82.08	44.05	97.71	0.54
Mite	95.29	95.80	90.81	68.48	99.02	0.76
Grass	87.81	87.91	87.16	49.27	98.25	0.59
Tree	82.10	81.56	86.88	35.85	98.14	0.48
Fungi	80.50	80.82	78.17	35.77	96.51	0.44
Other	82.50	83.62	61.13	17.29	97.55	0.26

### Comparison with existing methods

To benchmark the performance of the profile-based prediction methods, the five testing datasets, each consisting of 302 allergen sequences and 2000 non-allergen sequences, was used to evaluate six available techniques – the FAO/WHO evaluation scheme [5], SVM global description approach [8], SVM amino acid composition approach [14], SVM dipeptide composition approach [14], MEME motif discovery tool [12] and ARP technique [15]. The overall performance of each technique is indicated by the average performance over the five datasets.

As illustrated in Table 3, the overall performance of both general and group-specific profile-based models outperforms all other existing prediction systems investigated in this study. Both SVM amino acid and dipeptide composition methods [14] and the ARP technique [15] achieved high sensitivity (~89%) but low specificity (~57%) was also observed. The SVM global description approach [8] achieved the closest performance to the profile-based models in terms of accuracy (~93%). However, it exhibits high specificity (~95%) but low prediction sensitivity (~77%). The MEME motif discovery approach is shown to produce the lowest sensitivity (1.26%), which is lower than the reported sensitivity of 7% (at 0.001 E-value) [12]. This may be due to several reasons: i) differences in the testing dataset; and ii) the derived MEME motifs did not manage to capture essential features in allergen sequences. In agreement with previous reports [6,7], the FAO/WHO evaluation scheme predicts allergens with low specificity (23.31%) and low accuracy (31.58%). In contrast to PSSM, the FAO/WHO similarity-based evaluation scheme incorrectly predicts a large proportion of proteins derived from bacteria (37%), viruses (9%) and yeasts (9%) as positives. It is possible that some of these proteins may contain Ig-binding epitopes, though not necessarily demonstrate IgE binding. Among the false negatives, majority are distant homologues derived from fungi (39%), food (23%) and insect (9%).

**Table 3:** Comparison of the performance between the profile-based methods and existing allergenicity prediction systems

**Method**	**ACC (%)**	**SP (%)**	**SE (%)**	**PPV (%)**	**NPV (%)**	**MCC (%)**
General profile model	95.02	96.92	82.45	81.34	97.34	0.79
Group-specific profile model	94.88	96.52	84.04	79.49	97.56	0.79
FAO/WHO [5]	31.58	23.31	86.36	14.55	91.83	0.08
SVM (global description) [8]	93.01	95.40	77.22	59.02	96.52	0.71
SVM (aa composition) [14]	61.77	57.61	89.33	24.14	97.28	0.32
SVM (dipeptide composition) [14]	61.73	57.55	89.40	24.12	92.29	0.32
MEME/MAST motifs [12]	86.84	99.75	1.26	31.59	87.00	0.04
ARP [15]	61.55	57.45	88.74	18.92	97.12	0.31

## Conclusion

It is shown that profile-based methods are highly promising for assessing potential allergenicity and cross-reactivity in proteins with sensitivities and specificities of over 80%. The strength of such models lies in its ability to detect distantly related protein homologues through the use of iterated profiles [17-19]. To date, the exact mechanisms of allergy remains unclear as the structural, functional or biochemical properties of allergens that leads to allergic responses have yet to be elucidated. The allergen profiles that are constructed in this study may also be used as a basis for identifying common amino acid residues or physicochemical properties that support allergenicity [20].

## Methods

### Dataset

**Figure 1:**
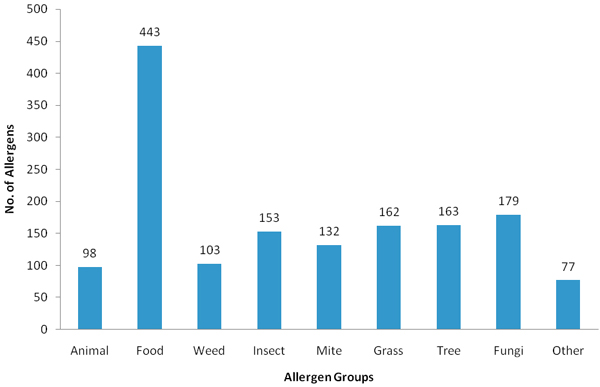
Distribution of the allergen data used in this study.

The training and testing dataset consist of 11,510 non-redundant (1,510 experimentally verified allergens and 10,000 putative non-allergens) sequences. Known allergen protein sequences were extracted from Swiss-Prot [23], GenBank [24], the Allergen Nomenclature database of the International Union of Immunological Societies (IUIS) [21], Allergome [25], the Food Allergy Research and Resource Program (FARRP) Protein AllergenOnline Database [7] and the Structural Database of Allergen Proteins (SDAP) [13]. The distribution of the allergen data used in this study is illustrated in Figure 1. An initial list of protein sequences unlikely to be associated with allergy was generated by extracting all protein sequences from Swiss-Prot with the exception of entries containing text strings 'allergen', 'allergy', 'atopy' or derivatives thereof in the annotation [9]. From this list, 10,000 putative non-allergens were randomly selected for model construction. Only 1 putative non-allergen sequence is extracted from each protein family to avoid bias.

The dataset was shuffled randomly and partitioned into five sets for five-fold cross validation, each time using one set for testing and the remaining four sets for training. Each training set contains 1,208 experimentally determined allergens and 8,000 non-allergens while each testing set contains 302 experimentally determined allergens and 2,000 non allergens.

### Profile-based methods

**Figure 2:**
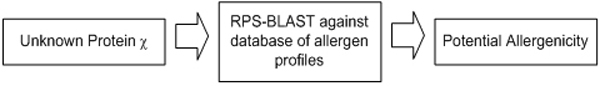
**General strategy of the profile-based method**. The general strategy involves performing a RPS-BLAST search on the query protein against a searchable database of allergen profiles generated by PSI-BLAST. Query sequences that generate hits above the specified e-value threshold are predicted to be potential allergens.

The general strategy of our iterative profile-based methods is shown in Figure 2. Allergen profiles are generated and optimized using sequences in the training set while sequences in the testing set are used to evaluate the overall performance of the profile-based methods. The system is implemented using the NCBI BLAST package [17] and PERL scripts.

#### Method 1: general allergen profiles

**Figure 3:**
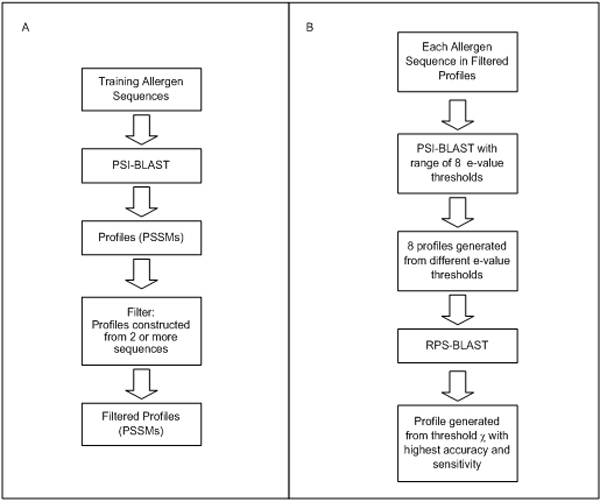
**Schematic representation of how allergen profiles are constructed in this study**. The development of this approach consists of A) a preliminary screening step and B) an optimization step.

This method predicts potential allergens by performing a RPS-BLAST search against a database of general allergen profiles optimized for accuracy and performance. The construction of allergen profiles involves an initial screening step and a subsequent optimization step, as outlined in Figure 3.

During the initial screening step, a PSI-BLAST search (10 iterations, e-value threshold 10^-3^) was performed on each allergen sequence in the training set against all other allergen sequences in the dataset. This generates a profile or PSSM for each allergen protein sequence. In this study, a minimum of two sequences was used for constructing a profile.

In the optimization step, another round of PSI-BLAST search was performed on each of the selected allergen sequence using eight different e-value thresholds (10, 1, 10^-1^, 10^-2^, 10^-3^, 10^-4^, 10^-6 ^and 10^-9^). This generates eight profiles for each allergen sequence corresponding to the different e-value threshold. Each of the eight profiles was tested by RPS-BLAST using allergen sequences in the training set as query. For each allergen sequence in the training dataset, the best profile (with the highest accuracy) was selected and incorporated into the predictive model. This approach produces a collection of general allergen profiles optimized for accuracy and performance.

#### Method 2: group-specific allergen profiles

This method predicts protein allergenicity by performing a RPS-BLAST search against a database of group-specific allergen profiles optimized for accuracy and performance.

Allergen sequences in the training set were partitioned into nine groups – i) weeds, ii) fungi, iii) grasses, iv) trees, v) mites, vi) animals, vii) insects, viii) food, and ix) others, according to the recommendation by the IUIS Allergen Nomenclature Sub-Committee [24]. For the screening phase, PSI-BLAST was performed by partitioning allergens into the 9 major groups and using individual groups of allergens as the training dataset. This generates profiles specific to each particular group of allergens, which are subsequently optimized according to their predictive accuracy and used for constructing group-specific allergenicity prediction systems.

### Performance measures

The predictive performance of the general and group-specific models was evaluated using sensitivity (SE), specificity (SP), accuracy (ACC), positive predictive value (PPV), negative predictive value (NPV), and Matthews correlation coefficient (MCC) [26]. In the latter, the positive dataset consists of testing allergen sequences belonging to a specified group whereas the negative dataset consists of all other allergen sequences in the testing set except the selected group. SE = TP/(TP+FN), SP = TN/(TN+FP) and ACC = (TP+TN)/*N*, indicate percentages of correctly predicted allergens, non-allergens and all proteins respectively. PPV = TP/(TP+FP) and NPV = TN/(TN+FN) denote the proportion of allergens and non-allergens that are correctly predicted, respectively. TP (true positives) represents known allergens and TN (true negatives) for non-allergens. FN (false negatives) denotes known allergens predicted as non-allergens, and FP (false positives) represents non-allergens predicted as allergens. The MCC, which is used to measure the randomness of the prediction, is computed and defined as follow:

$$MCC=\frac{\left(TP\times TN)-(FN\times FP\right)}{\sqrt{\left(TN+FN)(TP+FN)(TN+FP)(TP+FP\right)}}$$

The MCC returns a value between -1 and 1: MCC = 1 for 100% agreement of the prediction, MCC = 0 for completely random prediction and MCC = -1 for 100% disagreement of the prediction.

### Five-Fold cross validation

Five-fold cross validation was performed to assess the quality of all predictive models described in this study [20]. In *k*-fold cross-validation, *k *random, (approximately) equal-sized, disjoint partitions of the sample data are constructed, and a given model is trained on (*k*-1) partitions and tested on the excluded partition. The results are averaged after *k *such experiments, and the observed error rate may be taken as an estimate of the error rate expected upon generalization to new data.

## Competing interests

The authors declare that they have no competing interests.

## Authors' contributions

MTT designed the study, while all authors performed the experiments and the analyses. All authors have read and approved this manuscript.
